# Ether and Chloroform: Their Employment in Surgery, Dentistry, Midwifery, Therapeutics, Etc.

**Published:** 1851-05

**Authors:** 


ART. V.—*Ether and Chloroform: their employment in Surgery, Dentistry, Midwifery, Therapeutics, etc.* By J. F. B. FLAGG, M. D., Surgeon Dentist, Member of the Rhode Island Medical Society. Philadelphia: Lindsay & Blackiston. 1851.

Dr. Flagg has performed an acceptable service by this monograph on Anesthesia, and has presented the claims of the subject in a clear, forcible and philosophical manner. In 1849 the writer of this thought there was too great a disposition to resort to anesthesia in season and out of season, and an admonitory paper was published in this Journal on the subject. There seems to be now a current setting in from the opposite quarter, and there is something more than a salutary timidity in relation to the agents of anesthesia. The work of Dr. Flagg is well calculated to teach correct views, and to lead to proper conclusions.

Dr. Flagg's book treats of the following subjects: Oxide of Ethyl, or Ether, Chloroform, its Chemical Constitution, Ether, Review of the History of its Inhalation, General Directions for Administering Ether, etc., Physiological Effects of Sulphuric Ether, Sulphuric Ether in Surgery, Pathology, considered in connection with Ether Inhalation, Diagnosis, assisted by Ether Inhalation, Neurology, New Theory suggested, etc., Psychology, in connection with Ether Inhalation, Chloroform, as a Local Anesthesia, Anesthesia in Midwifery, Correspondence in Relation to the Efficacy of Ether and Chloroform, Miscellaneous Cases, Sulphuric Ether, and Unfavorable Impressions in regard to its use.

The following remarks are useful and judicious, and it is well for the inexperienced to have the advice contained in them, from such a discreet, experienced practitioner as Dr. Flagg. He says:

I know of no condition wherein the use of this article can be considered as really dangerous, if governed by a knowledge of its effects under all circumstances, and handled with that prudence which should ever be exercised in the use of all powerful agents. Steam may be used with *safety* in a poor boiler, in view of certain considerations, none of which, however, embrace the idea of ignorance or recklessness on the part of the engineer.

Ether, to be inhaled into the lungs, should be very highly rectified, washed free from its acid and alcoholic properties, rendering its specific gravity but little more than half that of distilled water.

No apparatus should be used as an *inhaler* which does not amply provide for the admission of atmospheric air, or that by any means would render the breathing difficult. A good sponge, well and frequently saturated with ether, is probably the best and simplest method of administering it; but a tumbler or cup for the purpose of holding a sponge, has been both convenient and economical, particularly when the frequent use of ether is required.

It is well to establish that understanding between yourself and your patients which will secure their most implicit confidence, both in yourself and in the full power of the ether to accomplish the end desired, requiring them to answer any question you may put to them while inhaling, as long as they perfectly comprehend you. By the *manner* in which they answer, you will find one very good guide when to perform the simple operation of extracting one or two teeth. I have found that partial etherization has been preferable in nearly all short operations, and particularly where the coöperation of the patient has been at all desirable.

The pulse should always be consulted. Its general tendency is upward or quickened. When this takes place very rapidly, it is proper to cease giving the ether for a few inhalations, or until reaction takes place, when a repetition of the ether will not unfrequently promote its downward tendency. Under ordinary circumstances I consider it safe to increase a pulse to 160 (one hundred and sixty) beats in the minute; or to suffer it to decrease to 50 (fifty), but of course there must be exceptions to this rule too obvious to need particularizing.

The expression of the countenance and temperature of the head should be observed. When the face becomes suddenly turgid, cold water should be applied to the forehead and temples, suffering the patient to breathe ammonia, or concentrated vinegar, allowing at the same time plenty of fresh air, directing them to take full and rapid inspirations. The variation of the temperature of the head is best detected by the habit of taking the pulse from the temporal artery.

The ether should be brought gradually to the mouth of the patient,—by so doing all irritation of the larynx and lungs is avoided. Direct to take regular and natural inspirations, to avoid swallowing the vapor; and should the breathing become stertorous, or in any way distressed, desist from its use until the natural breathing is restored.

If it should be found desirable to give the ether to that extent to induce narcotism, never suffer yourself to be thrown off your guard by any demonstration on the part of your patient. Firm, cool conduct on your part is more remarked by the patient than the casual observer would suppose. It is not unfrequent that a loud outcry may be induced, a distressing groan, or other manifestations of inconvenience or pain, which invariably pass off, and the strongest assurances are given by the patient, if aware of this fact, that he knows of no particular reason for such conduct.

It will be found, after inhaling for a minute or two and no effect is produced, that by allowing the patient to take one breath of atmospheric air considerable dizziness is felt. As a general thing, a very few inhalations after this are sufficient; and if the patient should resist, it is best not to meet that resistance by any physical force, but by firm yet kind treatment.

Should the operation be one that will require the introduction of the finger or thumb between the teeth of the patient, it may be well to protect it with a bandage, in view of his closing the mouth.

If ether should produce much prostration (which it is most apt to do when the atmosphere is least charged with oxygen), it is well to recommend self-exertion, as walking about, &c.; but if the desire to sleep is too strong to be resisted, entertain no fear from that indulgence, as the patient will soon awake refreshed.

After inhaling for a certain period, should any spasmodic action occur to retard breathing, sprinkling a little cold water suddenly on the face, or a pretty active slap between the shoulders, will be sufficient to relieve any obstacle in this respect.

I have occasonally given the ether for the removal of a tooth, and, when the effect passed off, some little complaint of headache was made, but a few inspirations of the same by the nose will invariably remove this difficulty.

The foregoing directions were prepared by myself in 1847, and published in the "Dental News-Letter," for the purpose more particularly, of securing a safe administration of ether in dental operations. When used, however, for painful and long-continued surgical cases, it should be given more intensely. The knife should not be used until the patient is brought completely under its influence; and fresh ether and fresh air should be alternately inhaled in such proportions as the case may require to keep the patient perfectly passive.

When used in labor, the directions are so very simple as to render it almost impossible that any one can be injured by it, even if she should take into her own hands its supervision. The pains are of a character that may always be entirely relieved or much mitigated by the ether, at a point far short of the loss of consciousness; and no woman of ordinary judgment could be using the ether for a space of five minutes, under these circnmstances, without really knowing more about it than any one can tell her, though a volume be filled in so doing. It is only necessary that the cloth or sponge should be frequently supplied with fresh ether. I would be understood in this, of course, to have reference to a natural labor and healthy condition of the patient.

In regard to its use in complicated cases, it will often be necessary to administer it to its greatest extent, and in view of this, those directions which are given for its use in dentistry and surgery will be found applicable.

*I believe there can be no previous condition of health which can be made to suffer by a judicious use of ether in childbirth, so much as by withholding it, unless, indeed, it be aneurism of the aorta;* and even to this critical condition of the system do I carry my doubts.

And in connection with this subject, we republish the following conclusions of M. Longet:

1st. In etherized animals there is absolute momentary suspension of sensibility, as well in all parts of the cerebro-spinal axis, usually sensitive, as in the nervous trunks themselves.

6th. The action of ether on the nervous system is much more directly and completely stupifying than alcohol, which merely renders the sensibility more obtuse, without suspending it entirely, at least in the nervous centres.

7th. Ether abolishes momentarily, but completely, the excito-motor, or reflex action of the spinal marrow and medulla oblongata; and, consequently, acts in an opposite manner to strychnine and opium, which exalt it.

9th. The functions of the encephalic centres always are suspended before those of the spinal marrow, and return before them.

10th. Ether supplies a new means of isolating, in the living animal, the seat of general sensibility from the seat of the intellect and will.

11th. In animals, we can so graduate the action of ether as to produce, at will, two stages, which I name, 1, *Etherization of the cerebral lobes*, 2, *Etherization of the annular protuberance.*

13th. Ether is only preventive of pain when it acts on the annular protuberance.

14th. In animals which have suffered etherization of the annular protuberance, this organ always recovers its functions as the perceptive centre of tactile impressions, before it becomes itself a sensible organ.

15th. The course of the phenomenon of etherization is far from being the same in men as in animals.

16th. The process of *de-etherization* of the annular protuberance may begin while *etherization* of the cerebral lobes still continues. This explains the cries that take place towards the end of some operations, which commence amid the most perfect quiet; cries, however, of which the patient retains no recollection on awakening.

17th. The *true chirurgical period* corresponds to that of *etherization of the annular protuberance* or absolute insensibility.

18th. For some time after the faculty of sensation is restored in etherized animals, there is transient exaltation of the sensibility.

20th. At a particular period of the experiments, the blood becomes almost black in the arteries. *Insensibility* always *shows itself previous to this occurrence.*

21st. If, after the point of total insensibility, inhalation is continued, the animals (rabbits), *cæteris paribus*, die within the space of from six to twelve minutes.

22d. On the contrary, on mixing a greater quantity of air with the vapor, the period of insensibility may be kept up a long time (three-quarters of an hour and more) without injury to the animal's life.*

23d. Ether introduced into the stomach does *not* produce insensibility in animals.

24th. In etherization, the functions of the *ganglionic nervous system* appear to be over-excited, and this system appears to become a sort of *diverticulum* for the nervous power which has, for the time, abandoned the *cerebro-spinal system.*

25th. The death of etherized animals is, perhaps, owing to a sort of asphyxia, originating particularly in the respiratory nervous centre.

The section on Psychology, in connection with ether inhalation, is full of interest; it has all the charm of a novel, and is something of a relief from the dry details of practice.

"Chloroform, as a local anesthesia," contains a number of very useful suggestions, for which the practitioner of medicine will find abundant use. We can bear testimony to the great value of chloroform as a local application; we have used it in a variety of pain, and it has scarcely ever disappointed our hopes. We are surprised to find no reference in Dr. Flagg's remarks to the combination of sulphate of morphine with the chloroform, for obtaining the complete anesthetic effects locally. The proportions are x. grs. morphine to ℥i. chloroform. The following cases are interesting:

A letter from my son contains an account of a case, of sufficient interest to justify the following extract:—"While in Valparaiso, I got a small, but long and hard thorn in my finger, at the root of the nail. I partially

---

* It would appear, from the above 21st and 22d propositions, that these experiments were carried on by means of an instrument calculated to exclude the atmosphere as much as possible. It was by such a course that the valuable horse in London, and the mouse alluded to in the "Lancet," were sacrificed to the cause of science.

extracted it, thinking I had it all; my finger festered, swelling much. I bathed it in lye-water, poulticing it, and was fearful of felon; but seeing a black speck, I attempted laying the finger open; the pain was excruciating on account of inflammation, but as soon as I brought blood, I applied a drop or two of chloroform. I then laid it open nearly to the bone, and about one inch in length, taking out even more of a thorn than at first, *and not feeling as much pain as I should had I operated upon some other person!* I then bandaged it, and allowed it to heal in the blood. At the end of the week I removed the bandage; the finger was well, and I saved the nail. *What was the value of that chloroform to me?*"

Dr. Isaac Hays reports a case of neuralgia of the foot, before the College of Physicians, Philadelphia. It is found in the "*American Journal of the Medical Sciences*," p. 550. Dr. Hays stated, "that he had employed the chloroform to produce local anesthesia, with apparently the most happy effects, in a case of neuralgia, occurring in a gentleman 50 years of age, who had been for a long time a sufferer from neuralgia of the foot, in which all the remedies that had been previously employed, failed to produce relief. Dr. H. was called to this patient about eight days since, and found him in intense pain, which had deprived him of sleep the whole of the preceding night. Dr. H. directed the affected parts to be enveloped with a pledget of lint, or a few folds of muslin wet with chloroform, and the whole to be covered with a portion of oiled silk, to prevent evaporation; on the next morning he found him entirely free from pain, which has not since returned. Whether the relief experienced in this case is to be ascribed to the local anæsthesia produced by the chloroform, or is to be considered as a mere coincidence, Dr. H. does not pretend to decide."

"Since this communication was made to the College, the further history of this case has shown that an arrest of the paroxysm is always accomplished by the application of the chloroform; and by the use of the article, several other similar cases have been attended with like results."

The following case illustrates the value of chloroform thus used: A lady for whom I had filled a large number of teeth, was much troubled by an abscess forming at the root of one of the incisors. It would occasionally become very painful; swell and discharge; when relief would ensue for several weeks. I had frequently treated this case with much care, till finding all accustomed remedies failing, and the annoyance becoming insufferable, the patient concluded to have it extracted. I stated to her that the local application of chloroform might give her one more chance of retaining her tooth, if used at a proper moment. I chose for that time, the earliest symptom of disquiet in the region of disease. The result has been immunity from pain, and the tooth is restored to its former usefulness. It has now been necessary to pursue this treatment but four or five times, at periods varying from one to two months, and the gums present a healthy appearance intermediately.

The section on anesthesia in midwifery contains several very interesting reports of cases. We believe that when ether or chloroform is judiciously administered to a female in labor, in the recumbent posture, on an empty stomach, there is almost, if not quite, a complete absence of danger. We have used chloroform many times, in cases of labor, since the appearance of Dr. Beatty's paper on the subject, in the *Dublin Medical Journal*, and we have not seen one untoward event in any of the cases. We quote from Dr. Flagg's work, the following letter from Mr. Lansdowne, of Bristol, England, to Professor Simpson:

I have now used ether or chloroform in seventy-one midwifery cases; I have two modes of administering it; the one with a bladder, in which is placed a brass pipe with a stop-cock, and into this is screwed, after I have poured the chloroform into the bladder, a piece of elastic tubing with a mouth-piece, the whole being pierced with a bore three-eighths of an inch, through which the vapor can be readily inhaled. If I find I am likely to be giving the chloroform for a long time, I use this apparatus, both for the sake of convenience and also of economy, as ʒj will last me nearly or quite an hour with this; and should

I use it many hours, it not only effects a great saving of material, but does not so frequently require replenishing, and is always ready at the approach of each separate uterine action, and it may be (as has been the case with me) used by any friends, or by the nurse, should the practitioner require to be absent for a short time. The other apparatus is an inhaler, such as is commonly sold; it is made of a thin, pliable lead, adapted over the nose and mouth, having a piece of perforated zinc in its front, and containing a piece of sponge, over which the chloroform is thrown; the depth of this inhaler is such as to prevent the nose being touched by the chloroform. It is home manufactured, not expensive, and very easy of construction. This latter I make use of if I am likely to be wanting it for a short time only; it requires to be supplied afresh every five or ten minutes, and accordingly I use ʒj or ʒss, which latter is my quantity when about to extract a tooth. If the action of the uterus causes great pain, as is frequently the case in an early period of the labor with the first child, I commence giving it as soon as the os uteri is sufficiently dilated for the head to pass; I have given it when the opening has not exceeded the size of half-a-crown. I believe it may be given with impunity as early in the labor as we please, and the only obstacles to its being so used that I can see, are the inconvenience to the medical attendant, in being thus occupied with one patient for such a length of time, and also the very great expense which such a lengthened use of it must entail. On the patient's account, I can see no possible reason why it may not be used for a whole day, or even more; indeed, I cannot see why a limit should be set to the length of time in which it may be used. I have no doubt but that it will soon be the anodyne generally used at the latter stages of painful cancerous diseases. The greatest length of time in which I have used it has been 16½ hours, a fresh inhalation being made at every renewal of the action of the uterus; in other cases I have given it 11½ and 12 hours, and the only reason of the inhalation being limited to this time has been the cessation of the necessity for its use, namely, that the child has been born, otherwise it would have been continued until such event had taken place.

I have found that nearly all my patients have recovered very rapidly; most of those who have had children previously, have been astonished at the unusual rapidity of their recovery.

I find no difference as to the expulsion of the placenta and the subsequent discharge, when administering chloroform, to what takes place in the usual natural labor. I have, upon two occasions, used it for severe after-pains, pains so severe that their cries could be heard at a considerable distance; indeed, they appeared worse than the pains of actual labor; in both cases the pain was completely subdued by its use. Both these persons had determined not to avail themselves of the benefit of the chloroform during labor; neither did they, but they were delighted afterwards with its soothing effects. The former of these, I had long resolved to give it to for this express purpose; it was her thirteenth child; her labors have always been very rapid, scarcely any pain accompanying them, but no sooner has the child been born than her agony has been almost past bearing, the pain recurring at intervals for a fortnight. Upon this occasion I gave it to her three times within the first ten hours, and she had nothing to complain of afterwards.

As regards sickness, I have not found that symptom, except where fluid has been previously taken; on the contrary, if the patient has been sick, the chloroform has almost invariably checked it. The cramp I have not heard them suffer from whilst under its influence. I have never yet met with anything which has caused me to regret having used it.

No practitioner of medicine is prepared for the proper practice of his duties, who is not qualified to use anæsthetic agents upon proper occasions, and the necessity for such qualification is as great as for any other duty required in the practice of medicine. If it is proper to relieve suffering, it is equally proper and obligatory to relieve it without suffering, if possible, and it is infinitely of more importance that a medical man shall possess full knowledge of the uses of anæsthetic means, than of the use of instruments for lithotomy or lithotrity. He may have use for the first remedies ten thousand times where he would require the instruments once.

A few days since, we were called upon to lance a severe paronychia, in a delicate, sensitive female. The agony experienced in the operation is well known to those who have had a trial of it, and we determined upon the use of the chloroform. The patient readily acquired its influence, and the finger was laid open to the bone, without any more suffering on the part of the patient than if she had not been disturbed. She speedily recovered from the effects, and assured the writer, next morning, that she had not enjoyed such a refreshing sleep for a long time. One of the greatest merits of anæsthesia in these and similar cases, is the absence of those sufferings that were once considered unavoidable after the operations are over. The patient in this case has scarcely had any suffering since her finger was opened, and this is not the rule in paronychia generally.

A short time since, a negro man was thrown from a horse and very much injured. He was so filled with pain that he could not bear the least motion without screaming. The left arm was badly broken, and gangrene attacked the arm and destroyed all vitality nearly up to the shoulder joint. We were called upon to visit him in the country, with Dr. Young, for the purpose of amputating the limb. In order to change the patient's position, for the removal of the arm, we attempted to raise his body, but his agony was so great that we were compelled to desist. Dr. Jacob, who accompanied us to the country—and whose very curious case, under anæsthesia, was reported in the February number of this Journal—was requested to attend to the chloroform, and a few inspirations of it, on the part of the patient, enabled us to move him as we pleased, without any sensibility on his part. The chloroform was continued throughout the amputation ; the arteries, four in number, were tied, the stump dressed, and everything completed, without any knowledge on the part of the patient. We would not have been without the beneficial effects of chloroform in this case, for any consideration. We had abandoned surgery bofore the introduction of anæsthesia, but have resumed it, now that we can relieve without suffering.

But let no one consider that he may use anæsthesia indiscriminately ; and as we have said that no man is qualified to discharge his duty as a physician, who is not qualified to use anæsthetic agents, we add, that we know of no finer manual for their use than this little book of Dr. Flagg's. We cordially commend it to the study of the profession, and very sincerely thank the author for the publication. B.

